# Circadian rhythms in the pineal organ persist in zebrafish larvae that lack ventral brain

**DOI:** 10.1186/1471-2202-12-7

**Published:** 2011-01-13

**Authors:** Ramil R Noche, Po-Nien Lu, Lauren Goldstein-Kral, Eric Glasgow, Jennifer O Liang

**Affiliations:** 1 Department of Biology, University of Minnesota-Duluth, 1035 Kirby Drive, Duluth, Minnesota, 55812 USA; 2 Department of Biology, Case Western Reserve University, 10900 Euclid Avenue, Cleveland, Ohio, 44106 USA; 3 Hathaway Brown High School, 19600 North Park Boulevard, Shaker Heights, Ohio, 44122 USA; 4 Department of Oncology, Georgetown University Medical Center, 4000 Reservoir Road NW Washington, DC, 20057 USA; 5 Department of Systems Biology, Harvard Medical School, 200 Longwood Avenue, Boston, Massachusetts, 02115 USA

## Abstract

**Background:**

The mammalian suprachiasmatic nucleus (SCN), located in the ventral hypothalamus, is a major regulator of circadian rhythms in mammals and birds. However, the role of the SCN in lower vertebrates remains poorly understood. Zebrafish *cyclops *(*cyc*) mutants lack ventral brain, including the region that gives rise to the SCN. We have used *cyc *embryos to define the function of the zebrafish SCN in regulating circadian rhythms in the developing pineal organ. The pineal organ is the major source of the circadian hormone melatonin, which regulates rhythms such as daily rest/activity cycles. Mammalian pineal rhythms are controlled almost exclusively by the SCN. In zebrafish and many other lower vertebrates, the pineal has an endogenous clock that is responsible in part for cyclic melatonin biosynthesis and gene expression.

**Results:**

We find that pineal rhythms are present in *cyc *mutants despite the absence of an SCN. The arginine vasopressin-like protein (Avpl, formerly called Vasotocin) is a peptide hormone expressed in and around the SCN. We find *avpl *mRNA is absent in *cyc *mutants, supporting previous work suggesting the SCN is missing. In contrast, expression of the putative circadian clock genes, *cryptochrome 1b (cry1b) *and *cryptochrome 3 (cry3)*, in the brain of the developing fish is unaltered. Expression of two pineal rhythmic genes, *exo-rhodopsin *(*exorh) *and *serotonin-N-acetyltransferase *(*aanat2*), involved in photoreception and melatonin synthesis, respectively, is also similar between *cyc *embryos and their wildtype (WT) siblings. The timing of the peaks and troughs of expression are the same, although the amplitude of expression is slightly decreased in the mutants. Cyclic gene expression persists for two days in *cyc *embryos transferred to constant light or constant dark, suggesting a circadian clock is driving the rhythms. However, the amplitude of rhythms in *cyc *mutants kept in constant conditions decreased more quickly than in their WT siblings.

**Conclusion:**

Our data suggests that circadian rhythms can be initiated and maintained in the absence of SCN and other tissues in the ventral brain. However, the SCN may have a role in regulating the amplitude of rhythms when environmental cues are absent. This provides some of the first evidence that the SCN of teleosts is not essential for establishing circadian rhythms during development. Several SCN-independent circadian rhythms have also been found in mammalian species. Thus, zebrafish may serve as a model system for understanding how vertebrate embryos coordinate rhythms that are controlled by different circadian clocks.

## Background

Circadian rhythms are biological cycles in behavior, physiology, and biochemistry that occur approximately every 24 hours. These oscillations are present in almost every organism, from cyanobacteria to plants to humans [1]. All circadian rhythms are regulated by a timing system composed of intracellular clocks with periods of approximately 24 hours, environmental cues, and clock-controlled outputs. An important characteristic is that circadian clocks are able to drive output rhythms even in the absence of environmental cues. However, environmental influences such as light and temperature are required to entrain or re-set the circadian oscillators so that they stay in synchronization with the organism's surroundings.

Regulation of vertebrate circadian rhythms is best understood in mammals. The SCN, a group of neurons in the hypothalamus, is among the most intensely studied cellular site of circadian oscillators. Targeted bilateral lesion of the rodent SCN abolished circadian oscillations in activity and drinking rhythms [2]. Conversely, transplantation of a donor SCN into a host with a lesioned SCN restored circadian cycling [3]. Further, the new locomotor activity rhythms of the host matched the circadian cycle of the donor SCN, indicating that the SCN was the major influence on the phase of the restored rhythms [3]. The SCN controls rhythms in other tissues through a variety of mechanisms, including the secretion of peptide hormones and synaptic signaling [4]. For instance, rhythmic production of the circadian hormone melatonin in mammals is controlled almost entirely by a multi-synaptic pathway leading from the SCN to the cells of the pineal gland [5].

Although it is clear that the mammalian SCN is important in controlling many rhythms in numerous organs, mammals also have circadian oscillators in other tissues as well as rhythms that are SCN-independent [6,7]. For instance, the mammalian retina has an endogenous oscillator that controls local rhythms such as visual sensitivity and retinal melatonin synthesis [8]. This indicates that the mammalian circadian system may have similarities to the circadian systems of many lower vertebrates, which often have many oscillators and many sites of photoreception [9-11].

A structure anatomically equivalent to the SCN has been described in zebrafish embryos and adults [12-17]. However, the function of the zebrafish SCN in regulating circadian rhythms is unknown [18]. This question is particularly interesting as many different isolated zebrafish cells and organs, such as the pineal, eyes, heart, spleen, and kidney, have endogenous oscillators and circadian photoreceptors that are able to generate cyclic gene expression [18-21]. For example, in adult zebrafish the pineal organ contains an endogenous circadian oscillator that is sufficient to drive rhythms in melatonin synthesis as well as photoreceptive neurons that entrain this oscillator [21]. However, whether pineal rhythms are also influenced by signals from other tissues, such as the SCN, is unknown in zebrafish and many other lower vertebrates. Interestingly, in some avian species, pineal rhythms are controlled both by an endogenous pineal oscillator and by input from the SCN, raising the possibility that multiple tissues could regulate pineal circadian rhythms in zebrafish [11,22,23].

Here, we provide evidence that the SCN is not required for pineal rhythms in developing zebrafish. To do this, we took advantage of zebrafish *cyc *mutants, which have a mutation in one of three zebrafish *nodal *genes [24,25]. Lack of Cyc/Nodal signaling results in a complete absence of the hypothalamus, including the regions that give rise to the SCN, the retro-chiasmatic nucleus, and the infundibulum [17,24-26]. Consistent with this earlier work, we find that expression of the *avpl *gene, which is typically expressed in and around the SCN [27,28], is absent in *cyc *mutants. Despite this, expression of the putative clock component genes *cry1b *and *cry3*, as well as the structure and size of the pineal organ was indistinguishable between *cyc *embryos and their WT siblings. The phase of gene expression of two pineal rhythmic genes, *exorh *and *aanat2 *persisted in the mutants. However, semi-quantitative analysis suggested that the amplitude of the rhythms was slightly reduced. *aanat2 *mRNA levels maintained their rhythmic changes in *cyc *embryos placed in constant conditions. This suggests that transcriptional rhythms were not being controlled by the environment, but rather by an internal cellular clock, perhaps the endogenous clock within the pineal cells. Since *cyc *mutants never have an SCN, this work indicates daily rhythms within the developing zebrafish pineal can be initiated in the absence of the developing hypothalamus, including the SCN anlage.

## Results

### *cyc *mutants lack *avpl *gene expression in the ventral brain

**Figure 1:**
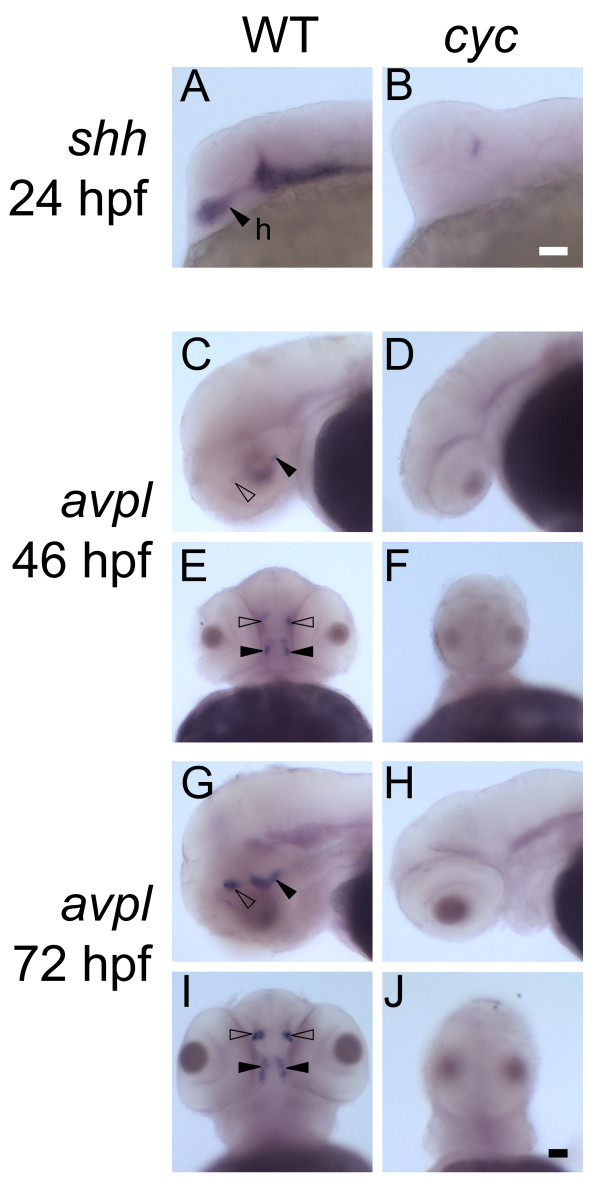
***avpl *expression is absent in *cyc *mutants**. Embryos were fixed at (**A, B**) 24 hours post-fertilization (hpf), (**C-F**) 46 hpf, and (**G-I**) 72 hpf and then processed for WISH with antisense probes for the indicated mRNAs. *shh-*expression in the ventral brain, including the hypothalamus (h, closed arrowhead) is (**A**) apparent in WT embryos but (**B**) absent in *cyc *mutants. (**C**, **E, G, and I**) *avpl *is expressed in the dorsal preoptic area (open arrowheads) and the ventral hypothalamus (closed arrowheads). (**D**, **F, H, and J**) Both *avpl *expression domains are eliminated in *cyc *mutants. (**A**-**D**, **G**, **and H**) are lateral views and (**E**, **F**, **I**, and **J**) are ventral views. The images in panels (**C**-**J**) are representative embryos of three independent experiments (n = 3 fish per experiment). *avpl *expression was also completely absent in *cyc *mutants at 47 hpf, Zeitgeber Time (ZT) 23.5; 52 hpf, ZT 5.5; 62 hpf, ZT 15.5, and 71.5 hpf, ZT 23.5. No circadian rhythm in *avpl *expression was detected in the WT larva processed in parallel (n>4 larva per time point). Scale bars = 50 μm.

We sought to define the function of the zebrafish SCN in regulating circadian rhythms during embryogenesis. Zebrafish *cyc *mutants are missing the ventral brain and the spinal cord, including expression of the genes *sonic hedgehog *(*shh*), *emx2*, and *nkx2.1 *in the hypothalamus [17,25,26,29]. This strongly suggests that *cyc *mutants could also be lacking the hypothalamus-derived precursors to the SCN. Consistent with previous data, we found that *cyc *mutants are missing the *shh*-positive neurons in the anterior hypothalamus that are proposed to be the precursors to the SCN (Figure 1A, B) [17,30].

To further characterize the ventral brain defect in *cyc *mutants, we examined the expression of the *avpl *gene. In mammals, vasopressin is expressed by the SCN neurons with a strong circadian rhythm, and has been linked to changes in hormone secretion from the pituitary, regulation of reproduction in females, and behaviors such as daily rhythms in wheel running activity in nocturnal rodents [28]. In non-mammalian vertebrates, vasopressin is replaced by Avpl, which is expressed in many ventral brain neurons including cells in and around the SCN [27]. In zebrafish, *avpl *mRNA is found in two domains of the ventral brain: the dorsal preoptic area and the anterior aspect of the ventral hypothalamus [31]. Based on the location of the *avpl *positive cells in the ventral hypothalamus, these cells likely correspond to the zebrafish SCN. In *cyc *embryos, both *avpl *expression domains were undetectable (Figure 1C-1J). This suggests that neurons expressing *avpl*, including those in the SCN, are missing.

### *exorh *mRNA is expressed rhythmically in the pineal organs of *cyc *mutants

**Figure 2:**
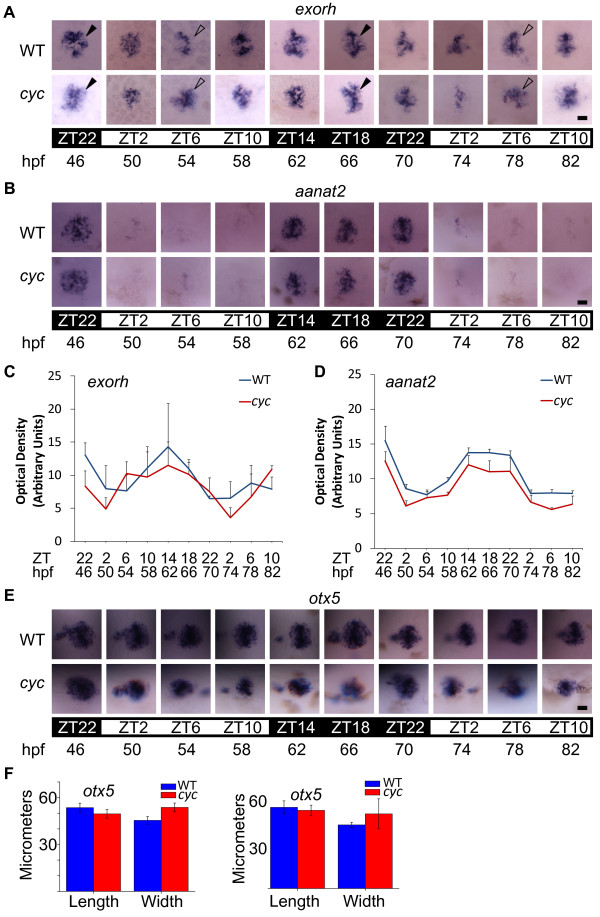
**Rhythmic expression of pineal genes persists in *cyc *mutants**. Embryos were raised in a 14:10 h L/D cycle and then sets of embryos were fixed at the indicated stages and ZT and processed for WISH for expression of (**A**) *exorh *or (**B**) *aanat2*. Note the time of peak (marked with closed arrowheads for *exorh*) and trough (marked with open arrowheads for *exorh*) expression is the same between both sets of embryos. (**C**, **D**) Quantification of the WISH signal indicates that the amplitude of *aanat2 *and *exorh *gene expression is slightly lower in *cyc *mutants than in their WT siblings. Two-way ANOVA reveals that the difference in the strength of the *aanat2 *signals are significantly different between *cyc *and WT (p < 0.01), while the difference in the strength of the *exorh *signals does not reach significance (p = 0.14). (**E, F**) In contrast, the expression of *otx5*, a gene expressed in all pineal cells, is comparable between WT and *cyc *mutants. (**F**) At 54 hpf, ZT6 (left graph) and 78 hpf, ZT6 (right graph), length of *otx5 *gene expression domain is similar between WT and *cyc *mutants, while the width of the pineal at each stage is slightly bigger in the mutants, perhaps due to changes in the morphology of the *cyc *head (54 hpf, pineal length, p = 0.11; 54 hpf, pineal width, p = 0.0045; 78 hpf, pineal length, p = 0.52; 78 hpf, pineal width, p = 0.14, n≥3 embryos per time point). In A, B, and E, light conditions are indicated by the white (light period) and black (dark period) bars. All images are dorsal views, anterior to the top. Each experiment was repeated three times, and representative images are shown. Scale bars = 20 μm.

We next wanted to examine the expression of rhythmic genes in a tissue outside of the SCN (Figure 2). The developing pineal organ was ideal for this analysis. The pineal contains an endogenous oscillator and photoreceptors that are able to entrain, or reset, this oscillator in response to photic input [21]. Further, pineal rhythms can be easily followed by assaying the expression of circadian-regulated pineal genes [32-39].

To determine what happens to pineal rhythms in *cyc *mutants, we first assayed the embryos for expression of *exorh *mRNA and protein. Exorh is a putative light-sensing, G-protein coupled receptor expressed in the pineal [40-43]. *exorh *mRNA is synthesized with a daily rhythm, with highest levels during the night and lower levels during the day [43,44].

Embryos were raised in a standard 14:10 h light/dark (L/D) cycle, with all other environmental parameters held constant. Rhythms in *exorh *mRNA were present in the pineal organs of both WT and *cyc *fish (Figure 2A, C). Further, the timing of the troughs and peaks of expression were similar between both sets of embryos (Figure 2A, C).

However, the amplitude of *exorh *expression was slightly lower at almost every time point tested in the *cyc *embryos (Figure 2C). To rule out the possibility that this was due to smaller pineal organs in the mutants, we measured the length and width of the pineal organs labeled for expression of *orthodenticle homeobox 5 *(*otx5*), a gene that is constitutively expressed in all pineal cells [35]. We found that the pineal size was not significantly different between WT and *cyc *fish (Figure 2E, F). This suggests that the decrease in the amplitude of *exorh *expression in *cyc *embryos was not due to smaller pineal organs.

### A gene involved in melatonin biosynthesis is expressed rhythmically in *cyc *mutants

*aanat2 *encodes the penultimate enzyme in the melatonin biosynthetic pathway [39]. In zebrafish and many other vertebrates, *aanat *genes are expressed in the pineal with dramatic differences between day and night levels [45]. Thus, *aanat2 *expression serves as a very sensitive readout of pineal circadian cycling. In WT and *cyc *embryos maintained in a L/D cycle, *aanat2 *transcripts cycled with indistinguishable periods and phases (Figure 2B, D). However, as was the case for *exorh*, the amplitude of *aanat2 *expression in *cyc *mutants was slightly diminished compared to WT siblings raised in parallel (Figure 2D). Thus, the nocturnal expression of *aanat2 *in the pineal was largely unaffected by the loss of the putative SCN in *cyc *mutant embryos.

### Rhythmic expression of *aanat2 *persists in *cyc *embryos placed in constant environmental conditions

**Figure 3:**
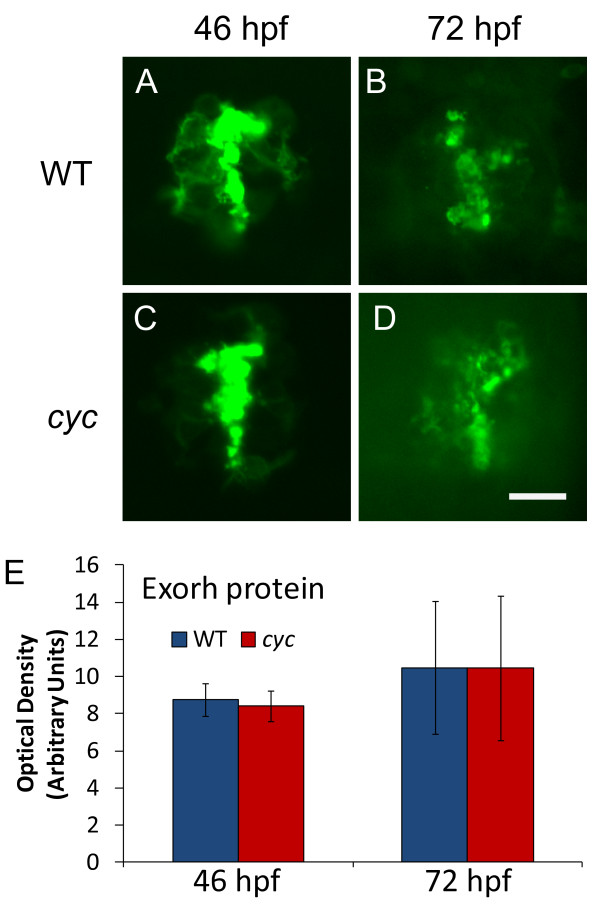
**Pineal Exorh protein expression is indistinguishable between *cyc *mutant and WT embryos**. Embryos were fixed and processed for whole mount antibody staining with the anti-Rhodopsin antibody 4D2. Quantification of fluorescent antibody signal shows that optical density of the pineal immunostaining is not significantly different at either time point (46 hpf, ZT 22, n = 10 embryos, p = 0.41; 72 hpf, ZT 0, n = 10 larvae, p = 0.67). Images are dorsal views with anterior to the top. Experiment was repeated two times with similar results, and representative embryos are shown. Scale bar = 20 μm.

**Figure 4:**
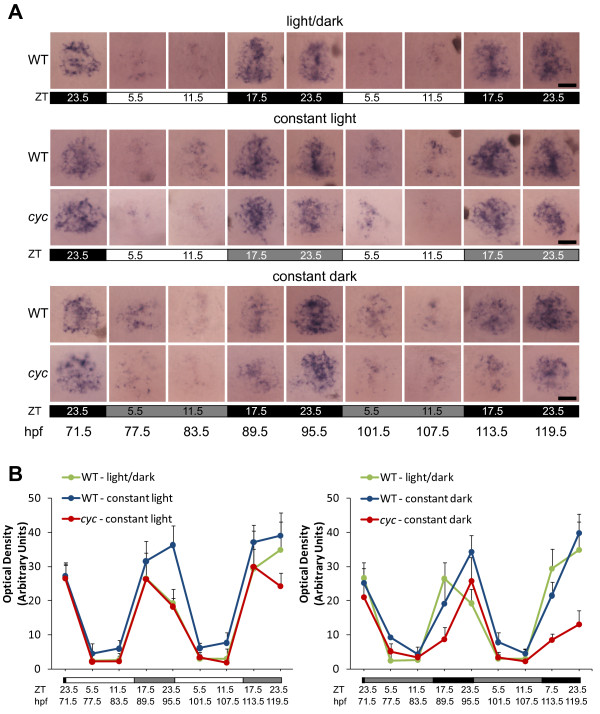
**Rhythmic expression of *aanat2 *persists in constant conditions**. Embryos were raised in a 14:10 h L/D cycle. At 71.5 hpf, ZT 23.5, a set of embryos was transferred to constant dark, constant temperature conditions. At 77.5 hpf, ZT 5.5 a set of embryos were transferred to constant light, constant temperature conditions. **(A) **Embryos were fixed at the indicated stages and ZT and processed for WISH for expression of *aanat2*. Note the time of peak expression is similar between the *cyc *embryos and their WT siblings. All images are dorsal views, anterior to the top. (**B**) Quantification of the WISH signal indicates that the amplitude of *aanat2 *gene expression is slightly lower in *cyc *mutants than in their WT siblings in constant conditions. Two-way ANOVA reveals that the differences between *aanat2 *signal is significantly different between *cyc *and WT (p < 0.01 for both constant light and constant dark, n>10 larva per time point, images of 4 larva per each experimental condition and time point used for statistical analysis). For the samples in a L/D cycle, position within the photoperiod is indicated by ZT and light conditions by the white (light period) and black (dark period) bars. For the constant light samples, the original L/D cycle is indicated by the ZT, and the white (original light period) and light grey (original dark period) bars. For the constant dark experiment, the original L/D cycle is indicated by the ZT, and the black (original dark period) and grey (original light period) bars. Experiment was repeated two times with similar results, and representative images from one of the experiments are shown in A. Scale bars = 20 μm.

The pineal organs of zebrafish, as well as other tissues, contain functional photoreceptors [18-21,46]. Further, we found that the Exorh protein was expressed normally in *cyc *mutants, suggesting that the pineal photoreceptors could be functional in these mutants (Figure 3)[Additional File 1]. Thus, one possibility was that the mRNA rhythms in the pinealocytes of *cyc *embryos were due to direct responses to changing light conditions, not to the presence of a circadian clock. To rule out this possibility, *cyc *embryos and their WT siblings were raised for two or three days in a standard L/D cycle, and then transferred to either constant light or constant dark for an additional two days (Figure 4)[Additional Files 2 and 3].

When the embryos were exposed to a L/D cycle for only two days before transfer to constant conditions, the amplitude of the rhythmic gene expression was very low in both WT and *cyc *mutants, suggesting that circadian cycling was not fully established by such a short period of entrainment [Additional File 2]. In contrast, when embryos were maintained instead for three days in a L/D cycle, rhythmic expression of *aanat2 *persisted in WT and in *cyc *larva after transfer to either constant light or constant dark conditions (Figure 4). Under constant dark conditions, a slight shift in the peak of expression to the right was apparent, consistent with previous studies that showed the endogenous period of the clock that controls zebrafish pineal rhythms is slightly longer than 24 hours (Figure 4B) [10,35,39,47]. However, this shift was not readily apparent in fish in constant light (Figure 4B). The persistence in cyclic changes in expression after transfer to constant lighting conditions suggests that the rhythms are not due to responses to rhythmic environmental changes, but instead due to a functioning circadian clock in the *cyc *embryos.

Although the overall rhythm in gene expression persisted in constant conditions, there were some significant differences between the *cyc *mutants and their WT siblings. As in the L/D experiments (Figure 2), the levels of mRNA were slightly lower in the *cyc *embryos at most time points (Figure 4). Further, after approximately 48 hours in constant conditions, for both constant light and constant dark, the *aanat2 *expression was notably lower in the *cyc *embryos than in their WT siblings (Figure 4).

### *cry *gene expression is present in *cyc *mutants

To gain insight into other potential changes in *cyc *embryos, we examined the expression of putative clock components during embryogenesis. The vertebrate circadian clock is composed of complex positive and negative feedback loops that take approximately 24 hours to go through one cycle. Cry proteins are essential components of this clock, in which they act as transcriptional repressors in the negative feedback loop. Zebrafish have six *cry *genes, all of which are expressed with a daily rhythm [48,49].

**Figure 5:**
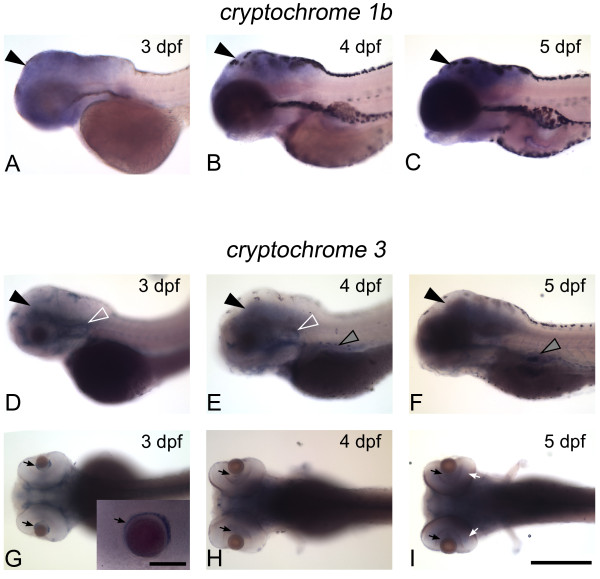
**Expression of *cry *genes in zebrafish larvae**. WT larvae were fixed at the indicated stages and then processed for WISH with an antisense probe for *cry1b *or *cry3*. (**A-C**) *cry1b *is expressed in the brain (closed arrowhead) from 3 to 5 dpf. (**D, G**) At 3 dpf, *cry3 *is expressed in the brain (closed arrowhead), ear (open arrowhead) and in a region of the retina around the lens (black arrows). Inset in panel G is a higher magnification of the area around the lens. (**E, H**) At 4 dpf, *cry3 *is expressed in the brain (closed arrowhead), ear (open arrowhead), and liver (gray arrowhead). (**F, I**) By 5 dpf, *cry3 *transcripts are present in the ganglion cell (black arrows) and inner nuclear layers (white arrows) of the retina, and persist in the brain (closed arrowhead) and liver (gray arrowhead). WISH at each stage was repeated two times and representative images are shown (n≥5 embryos for the experiment shown). Panels (**A-F**) are lateral views, anterior to the left and panels (**G-I**) are dorsal views, anterior to the left. Scale bar in panel I (for all images except G inset) = 500 μm, Scale bar for G inset = 100 μm.

**Figure 6:**
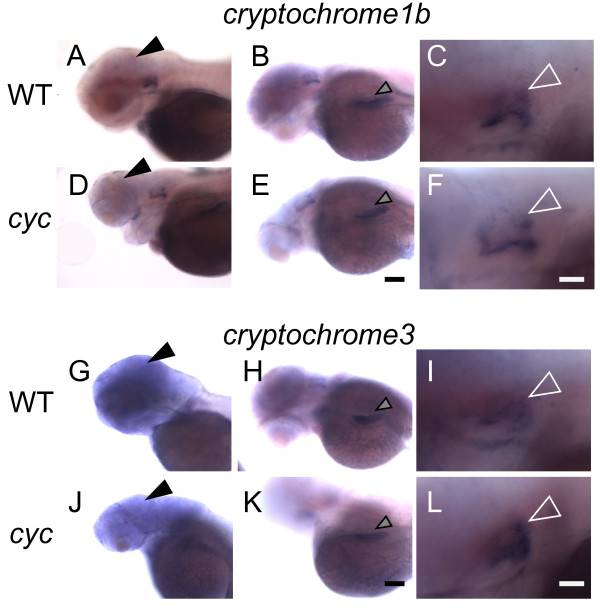
***cry *gene expression is present in *cyc *mutants**. *cyc *embryos and their WT siblings were raised in a 14:10 h L/D cycle, fixed at 72 hpf, ZT 0, and then processed for WISH. In both (**A**-**C**, **G**-**I**) WT embryos and (**D**-**F**, **J**-**L**) *cyc *mutants, *cry1b *and *cry3 *are expressed in the (**A**, **D**, **G**, **J**) brain (closed arrowheads), (**B**, **E**, **H**, **K**) liver (gray arrowheads), and (**C**, **F**, **I**, **L**) ear (open white arrowheads). Experiment was repeated three times, and representative images are shown. All images are lateral views, anterior to the left. Scale bars = 40 μm for (**A**-**B**, **D**-**E**, **G**-**H**, **J**-**K**) and 100 μm for (**C**, **F**, **I**, **L**).

We found that the expression of *cry1b *and *cry3 *was indistinguishable between *cyc *embryos and their WT siblings (Figures 5 and 6). In both types of embryos, *cry1b *was expressed widely in the developing brain (Figure 5). *cry3 *was also expressed in the brain, and more strongly in the ear, liver, and in retinal cells near the lens (Figures 5 and 6). These results indicate that transcription of *cry1b *and *cry3 *is present even when the ventral brain is absent.

## Discussion

In mammals and birds, the SCN has an important role in regulating circadian rhythms throughout the organism. Retinal photoreceptors entrain circadian oscillators within the SCN, and the SCN then regulates pineal rhythms through a multisynaptic pathway [50]. Although the retina-SCN-pineal pathway is present in fish, amphibians, and reptiles, its function is not well understood [15,51-53]. Here we provide evidence that pineal circadian rhythms are established and have normal phase in zebrafish *cyc *mutants, which lack ventral brain. This suggests that neither the SCN nor the retinal-hypothalamic pathway is essential for the onset of pineal circadian rhythms during embryogenesis. However, the amplitude of the rhythms is slightly, but significantly decreased, in *cyc *mutants. This suggests that the SCN or other tissues that are missing in these mutants, which include other regions of the ventral brain, may have a role in regulating rhythms during development.

### Pineal rhythms persist in *cyc *mutants

The SCN has been defined anatomically in zebrafish embryos and adults [15]. However, the function of the zebrafish SCN is unknown. We used *cyc *mutants to test the function of the zebrafish SCN. These mutants are missing the whole ventral brain including the entire hypothalamus, and thus, the precursors to the SCN [17,26]. We now demonstrate that expression of *avpl*, which is usually present in and around the SCN, is lacking in *cyc *mutants (Figure 1). This provides strong evidence that the SCN is completely absent in *cyc *mutants.

We found that rhythmic expression of *aanat2 *and *exorh *are present in *cyc *mutants during the first few days of development (Figure 2). The phases of expression of these genes are indistinguishable between *cyc *mutants and WT siblings raised in parallel in a L/D cycle. Further, the *aanat2 *expression rhythms persist after the embryos are transferred to constant dark or constant light, indicating they are being driven by a circadian clock (Figure 4).

### Diminished amplitude of gene expression in *cyc *mutants

The expression amplitude of *aanat2 *and *exorh *mRNA levels are slightly reduced in the *cyc *mutants compared to WT siblings raised in parallel. This was true for fish in a L/D cycle, and after transfer to constant dark or constant light conditions (Figures 2 and 4). In addition, the decrease in amplitude of expression became more pronounced when the fish approached the third day in constant conditions (Figure 4).

*cyc *mutants have many developmental defects, making it difficult to precisely identify the cause of the decrease in gene expression. It is unlikely that the change is due to defects in pineal development, as the size of the pineal was not significantly decreased in *cyc *mutants (Figure 2F). However, the lower amplitude of gene expression could be due in part to poor health or subtle changes in the pineal that we have not detected [24-26].

A more interesting possibility is that the SCN or other missing tissues in the ventral brain play a role in regulating the amplitude of pineal rhythms. Because the phenotype of *cyc *mutants becomes more severe as the fish get older, we kept the fish in a L/D cycle for only a short time before being assayed. Thus, one possibility is that the SCN/ventral brain functions to help establish self-sustaining rhythms during development. This role for the SCN could explain why the amplitude of rhythms were lower in the *cyc *pineal overall, and why the rhythms in the *cyc *pineal started to dampen sooner after transfer to constant conditions. Testing this hypothesis will require a mutant that more specifically affects the SCN or a way to specifically inhibit the function of SCN cells.

### Tissues that could be promoting circadian rhythms in *cyc *mutants

We do not completely understand what is driving pineal rhythms in *cyc *mutants. One possibility is that these rhythms are driven by clocks within the zebrafish pineal. Persistent circadian rhythms in melatonin synthesis were observed previously in isolated adult zebrafish pineals cultured for several days [21]. This suggests that the pineal oscillators in adult zebrafish are self-sustained. Clocks in the embryonic pineal may work in the same way. In support of this, a previous study showed that light input very early in development, long before the differentiation of an SCN, is necessary for onset of pineal rhythms [54].

Oscillator proteins present in the pineal are also in many other zebrafish cells and tissues. Likewise, circadian rhythms have been detected in isolated cultured zebrafish embryonic and adult tissues [18-20]. Thus, it is possible that other tissues present in *cyc *mutants are interacting to promote circadian cycling in the pineal.

We also cannot rule out a role for the lateral eyes. The first zebrafish retinal photoreceptors differentiate at approximately 2.5 dpf [55]. Thus, detection of environmental light conditions by the retina could explain why circadian rhythms in pineal gene expression were better established when embryos were maintained for three rather than two days in a L/D cycle (compare Figure 4 with Additional file 2). Previous work suggested that the lateral eyes are not required for establishing rhythms in the pineal. Rhythms in pineal *aanat2 *expression are present in zebrafish *chokh *(*chk*) mutants, which lack lateral eyes [56]. Further, *chokh *mutants can re-entrain to a shifted L/D cycle [56]. However, the persistence of pineal rhythms in *chokh *fish after transfer to constant lighting conditions has not yet been tested, raising the possibility that they could have defects similar to those we found in *cyc *mutants.

A final possibility is that pineal rhythms are present in *cyc *mutants because there are some residual SCN neurons. However, this is unlikely, as the whole ventral brain is absent in *cyc *mutants (Figure 1A-1B) [17,24-26,29]. *chk *mutants are missing both eyes and hypothalamic *avpl *expression [57]. Despite this, they have rhythms in gene expression of two clock components (*clock *and *period4*) and pineal *aanat2 *[56,58]. These results are consistent with our findings that loss of neurons expressing *avpl*, including those in the SCN, does not abolish pineal rhythms.

### Normal *cry *gene expression in *cyc *mutants

Cry proteins are required for regulation of circadian rhythms in both plants and animals [59]. These proteins were shown to play two major roles in regulation of circadian rhythms: as a photoreceptor to entrain the clock and as a repressor of Clock/BMAL-induced circadian transcription [59]. Six Cry proteins are present in zebrafish [60]. By heterologous expression, it was shown that Cry1b, but not Cry3, is capable of blocking Clock/BMAL-dependent transcription [60]. This suggests that Cry1b and Cry3 have different functions. We find that *cry1b *and *cry3 *mRNAs are found in specific tissues of zebrafish embryos and larvae (Figures 5 and 6). Further, we demonstrate that *cry1b *and *cry3 *expression in developing brain, ear, and liver are present in *cyc *mutants (Figures 5 and 6). Although the function of the *cry *genes in zebrafish is not yet fully understood, the presence of *cry1b *and *cry3 *transcripts in the *cyc *brain suggests that the circadian clock may not be severely disrupted.

### Exorh protein expression is normal in *cyc *mutants

*exorh *was originally identified as a rhodopsin class gene expressed in the zebrafish and salmon pineal organs [40,61]. Exorh has been predicted to be a G-protein coupled receptor with ~70% amino acid sequence identity with vertebrate Rhodopsin [40]. Our group and others have previously demonstrated the rhythmic expression of *exorh *mRNA [43,44]. In contrast to *exorh *mRNA, we find that Exorh protein expression does not follow a significant daily rhythm in WT embryos, nor is its expression affected in *cyc *mutants (Figure 3)[Additional File 1].

The functional significance of this difference in temporal expression pattern is unknown. There are other cases where mRNA displays cyclic expression, while the corresponding protein does not. In the chicken pineal gland, the mRNA encoding the photopigment Pinopsin shows a rhythm that is dependent upon activation by light [62]. In zebrafish, *interphotoreceptor retinoid-binding protein *(*irbp) *mRNA expression is circadian-regulated, while and IRBP protein levels are constant [37,63]. In the case of IRBP, it is proposed that the higher expression of mRNA during the day is necessary to maintain the constant levels of protein, as protein turnover is much higher during the day than during the night [37,63]. This could be the explanation for the rhythmic pattern of *exorh *and *pinopsin *mRNA expression as well. Consistent with this, Pinopsin protein and mRNA levels decline rapidly when the embryos are placed in constant darkness [62].

## Conclusion

More than two decades ago, a structure anatomically equivalent to the SCN was first described in a teleost fish (the goldfish) [64,65]. Several years later, a morphologically similar group of neurons was described in zebrafish [15]. However, no experimental evidence exists for a pacemaker role of the SCN in zebrafish or in other teleosts [18]. Here, we establish that the zebrafish SCN is not required to establish circadian rhythms in the embryonic pineal. However, the amplitude of circadian gene expression was slightly reduced, and so we cannot rule out a role for the SCN or other tissues missing in *cyc *mutants in helping to establish or maintain rhythms in developing zebrafish. This will form a strong foundation for future studies that explore the communication between different circadian tissues during development, and for comparative studies of pineal biology and circadian regulation between vertebrate species.

## Methods

### Zebrafish

Zebrafish embryos and larvae were obtained by natural matings and were raised at 28.5°C at 14:10 hour (h) L/D cycle according to standard procedures [66]. Developing fish were placed in a Sanyo MIR-153 incubator (Amsterdam, The Netherlands) with heating and cooling capabilities for maximum temperature stability. Light consisted of a single 60 watt Globe EnerSaver light bulb placed within the incubator. Stocks used were Oregon AB (WT), ZDR (WT) (Aquatica Tropicals, Plant City, FL), and *cyc*^m294 ^[25,66-68].

### Whole mount RNA in situ hybridization (WISH)

WISH was carried out as described by Liang et al. [69]. Antisense RNA probes included *aanat2 *[39], *exorh *[40], *shh *[69,70], *cry1b *[49], *cry3 *(Open Biosystems, Huntsville, AL), *otx5 *[35], and *avpl *[31].

To generate *cry1b *probe, *cry1b *cDNA in plasmid pME-18S-FL3 was subcloned into plasmid pBSK(+) using a 5' EcoRI and a 3' NotI restriction sites. Plasmid pBSK+ *cry1b *was linearized with EcoRI and was used for probe synthesis with T3 RNA polymerase. *cry3 *probe was PCR amplified from the *cry3 *cDNA in plasmid pME-18S-FL3 using primers and cycling conditions in ZFIN [http://zfin.org/cgi-bin/webdriver?MIval=aa-markerview.apg&OID=ZDB-CDNA-040425-188]. *cry3 *mRNA was made with T3 RNA polymerase.

### Whole mount antibody staining

Whole mount antibody staining was done as described [71]. A mouse monoclonal antibody (4D2) against the N-terminus of bovine Rhodopsin [72,73] was used to detect Exorh protein at a dilution of 1:60. A goat anti-mouse secondary antibody coupled to Oregon Green 488 (Invitrogen Molecular Probes, Carlsbad, CA) was used at a dilution of 1:2000.

### Morpholino and mRNA injections

Control and translation blocking morpholinos (MO) against *exorh *(exorh atg MO) were obtained from Gene Tools (Philomath, OR). Sequences of the MO used were: control MO, 5'-CCT CTT ACC TCA GTT ACA ATT TATA-3' and exorh atg MO, 5'-AGT TGG GTC CCT CCG TCC CGT TCAT-3'. One-cell stage embryos were injected with either 1.5 nanograms (ng) of control or exorh atg MO using a Harvard Instruments PLI-90 Pico-Injector (Holliston, MA).

To generate *exorh *mRNA for overexpression, the entire coding sequence of the zebrafish *exorh *gene (from plasmid pCR2.1-full-length *exorh*) [GenBank Accession Number: AB025312] was subcloned into pGEMHE plasmid [40,74]. Donor and host plasmids were digested with EcoRI. The resulting EcoRI-digested full-length *exorh *coding sequence was ligated non-directionally into pGEMHE (plasmid pGEMHE-full-length *exorh *hereafter pGEMHE-flex) using the TAKARA DNA Ligation Kit version 1 (Madison, WI). The ligation reaction was transformed into Z-Competent *E*. *coli *cells (Zymo Research, Orange, CA). Clone orientation was verified via restriction digestion (double digestion with SacI and BanII) and DNA sequencing. DNA sequencing was done using forward and reverse sequencing primers (forward primer: 5'-TTT TTG CAG AAG CTC AGA ATA-3'; reverse primer: 5'-CAT TTT GTA AAG TGT AAG TTG GTAT-3'). DNA sequences were verified by doing a BLAST search [75].

To synthesize full-length *exorh *mRNA, pGEMHE-flex plasmid was linearized with SphI. Linearized pGEMHE-flex was used for mRNA synthesis with an Ambion mMESSAGE mMACHINE kit (Austin, TX). Full-length *exorh *mRNA was verified through RNA formaldehyde agarose gel-electrophoresis. One blastomere of 8-16 cell stage embryos were injected with 400 picograms (pg) of beta-galactosidase (control) or full-length *exorh *mRNA and were fixed 7 h post-injection. Injected and fixed embryos were processed for whole mount antibody staining as described above.

### Photography and Image Analysis

Embryonic and larval zebrafish samples were imaged using a Zeiss Axioplan 2 Imaging Microscope (Thornwood, NY) or Nikon Eclipse 801 Epifluorescent Microcope (Melville, NY) connected to a Spot RTke7.4 Slider digital camera together with Spot 4.5.9.1 software (Diagnostic Instruments, Sterling Heights, MI). Resulting images were processed and quantified for optical density using ImageJ 1.36b and 1.42q software [Rasband, W.S., ImageJ, U. S. National Institutes of Health, Bethesda, Maryland, USA, http://rsb.info.nih.gov/ij/, 1997-2009]. Calibration was performed following developer's instructions [http://rsb.info.nih.gov/ij/docs/examples/calibration/]. For in situ hybridization data, images were first converted to 8-bit grayscale and an oval encompassing the expression domain was drawn using the Specify tool. Optical Density (OD) was calculated by multiplying the oval area with the average intensity, both of which were obtained through the Analyze tool. Two-way Analysis of Variance (ANOVA) statistical analysis was carried out using Microsoft Excel.

For quantification of *aanat2 *expression in the constant light and constant dark experiments, the background value was subtracted as follows. A small circle with diameter of 20 pixels was selected close to, but not touching the anterior edge of the WISH expression signal. The circular area and optical density was determined as above. Background-corrected OD values were calculated by subtracting the (OD value of small circle × pineal oval area/small circle area) from the OD of oval around the pineal expression domain. All OD data are means ± standard deviation. Representative images with ODs closest to the mean were chosen for figures.

Fluorescent images were analyzed as above with the exception that 8-bit grayscale images were first processed using the Inverted tool prior to choosing the oval area to be quantified for OD. For Exorh protein temporal expression, fluorescent image data was tested for significance using One-way ANOVA and Tukey's test using Origin Software Version 7.5 SR4 (Northampton, MA).

To measure the dimensions of the *otx5 *expression domain, lines covering the length or the width of the pineal were drawn using ImageJ and the corresponding pixel length or width were converted to micrometers by calibrating the number of pixels in a 20 micrometer line.

## Authors' contributions

RRN conceived of and designed the study, performed experiments on Exorh protein expression and antibody staining, and studies on *aanat2 *rhythms, *shh, cry*, and *otx5 *expression in *cyc *mutants, and wrote the first draft of the manuscript. PL helped optimize the antibody staining protocol and carried out studies on *cry1b *and *cry3 *expression during development, *exorh *mRNA rhythms in *cyc *mutants, and parts of the constant light and constant dark experiments. LGK assisted in designing and in performing the experiments on *aanat2 *rhythms and *otx5 *expression in *cyc *mutants raised in L/D. EG cloned and generated the probe for *avpl *and performed WISH on *cyc *mutants. JOL helped design and coordinated the study, carried out parts of the constant light and constant dark studies and the *avpl *in situs. All authors helped write the manuscript and have approved the final version.

## Supplementary Material

Additional File 1 **Exorh protein is expressed without a significant rhythm **(**A, B**) Embryos were injected with (**A**) control or (**B**) exorh atg MO, fixed at 64 hpf, and processed for fluorescent whole mount immunostaining with the anti-bovine Rhodopsin antibody 4D2. **(A) **Control embryos have robust fluorescent signal in the pineal organ that is (**B**) severely reduced in Exorh depleted embryos. (**C-C'**) Embryos injected with *beta-galactosidase *mRNA have undetectable levels of immunoreactivity with the 4D2 antibody at 8 hpf. (**D-D'**) In contrast, embryos injected with *exorh *mRNA show strong antibody staining at 8 hpf. (**E**, **F**) Embryos were fixed in a circadian time course and then processed for 4D2 antibody staining. One-way Analysis of Variance (ANOVA) and Tukey's analysis revealed no significant changes in pineal Exorh protein levels that followed a daily rhythm (n≥9 embryos per time point). However, a few time points were significantly different (p ≤ 0.05) from each other including 72 and 108 hpf, 72 and 116 hpf, 76 and 116 hpf, and 80 and 116 hpf. (**A**-**B, and E**) are dorsal views, anterior to the top and (**C**-**D'**) are lateral views. (**C'**) and (**D'**) are higher magnification images of the regions boxed in (**C**) and (**D**), respectively. Scale bars = 20 μm for (**A-B, C', D', E**) and 100 μm for (**C, D**). Click here for file

Additional File 2 **Two days in a L/D cycle is not sufficient to initiate robust circadian cycling of *aanat2 *expression**. Embryos were raised in a 14:10 h L/D cycle. At 47.5 hpf, ZT 23.5, a set of embryos was transferred to constant dark, constant temperature conditions. Embryos were fixed at the indicated stages and ZT and **(A) **processed for WISH for expression of *aanat2 *and **(B) **the WISH signal was quantified. Note the time of peak (closed arrowheads) expression is similar between the *cyc *embryos and their WT siblings. All images are dorsal views, anterior to the top. For the samples in a L/D cycle, position within the photoperiod is indicated by ZT and light conditions by the white (light period) and black (dark period) bars. For the constant dark samples, the original L/D cycle is indicated by the ZT and the black (original dark period) and dark grey (original light period) bars. Experiment was repeated two times with similar results, and representative images from one of the experiments are shown. Scale bars = 20 μm. Click here for file

Additional File 3 **Comparison between larva processed for WISH with antisense and sense probes reveals low background staining**. Embryos were raised in a 14:10 h L/D cycle and fixed and processed for WISH using *aanat2 *antisense or sense probe. Note that the sense probe produces no detectable signal, as it would recognize antisense mRNA, which should not be present. The brown regions are melanocytes in the skin, which have a natural pigment. All images are dorsal views, anterior to the top, with the pineal indicated (closed arrowheads). Position within the photoperiod is indicated by ZT and light conditions by the white (light period) and black (dark period) bars. Representative images are shown. Scale bar = 30 μm. Click here for file
