# Supplementary material for: Circadian rhythms in the pineal organ persist in zebrafish larvae that lack ventral brain
Source: BMC Neurosci. 2011 Jan 13;12:7. doi: 10.1186/1471-2202-12-7 (PMC3031267; doi:10.1186/1471-2202-12-7)
Supplement: Additional File 1 — Exorh protein is expressed without a significant rhythm (A, B) Embryos were injected with (A) control or (B) exorh atg MO, fixed at 64 hpf, and processed for fluorescent whole mount immunostaining with the anti-bovine Rhodopsin antibody 4D2. (A) Control embryos have robust fluorescent signal in the pineal organ that is (B) severely reduced in Exorh depleted embryos. (C-C') Embryos injected with beta-galactosidase mRNA have undetectable levels of immunoreactivity with the 4D2 antibody at 8 hpf. (D-D') In contrast, embryos injected with exorh mRNA show strong antibody staining at 8 hpf. (E, F) Embryos were fixed in a circadian time course and then processed for 4D2 antibody staining. One-way Analysis of Variance (ANOVA) and Tukey's analysis revealed no significant changes in pineal Exorh protein levels that followed a daily rhythm (n≥9 embryos per time point). However, a few time points were significantly different (p ≤ 0.05) from each other including 72 and 108 hpf, 72 and 116 hpf, 76 and 116 hpf, and 80 and 116 hpf. (A-B, and E) are dorsal views, anterior to the top and (C-D') are lateral views. (C') and (D') are higher magnification images of the regions boxed in (C) and (D), respectively. Scale bars = 20 μm for (A-B, C', D', E) and 100 μm for (C, D). [file 1471-2202-12-7-S1.PDF]

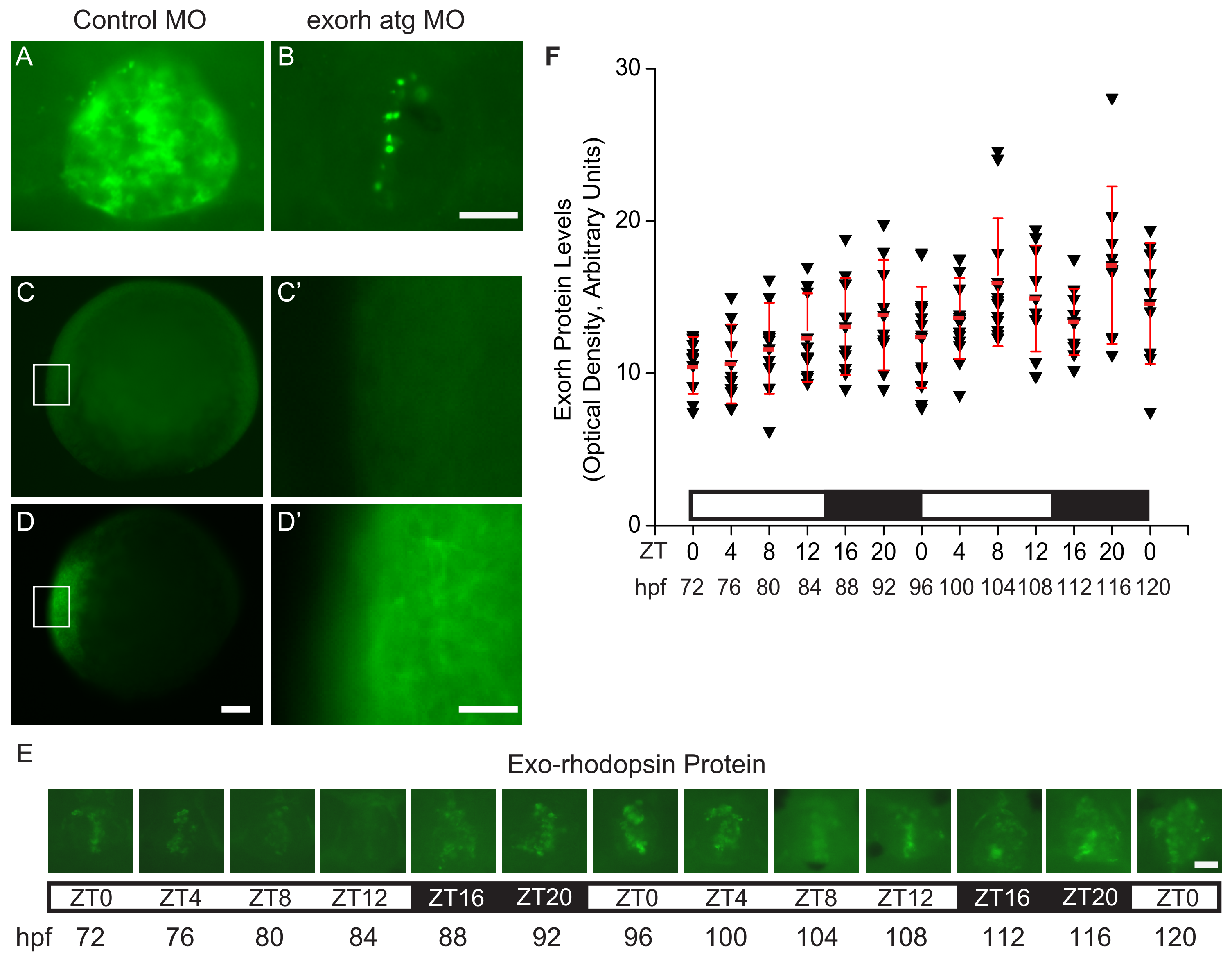

Control MO
exorh atg MO
A
B
C
C'
D
D'
F
Exorh Protein Levels
(Optical Density, Arbitrary Units)
30
20
10
0
ZT 0 4 8 12 16 20 0 4 8 12 16 20 0
hpf 72 76 80 84 88 92 96 100 104 108 112 116 120
E
Exo-rhodopsin Protein
ZT0 ZT4 ZT8 ZT12 ZT16 ZT20 ZT0 ZT4 ZT8 ZT12 ZT16 ZT20 ZT0
hpf 72 76 80 84 88 92 96 100 104 108 112 116 120
