# Supplementary figures and images for: Circadian rhythms in the pineal organ persist in zebrafish larvae that lack ventral brain
Source: BMC Neurosci. 2011 Jan 13;12:7. doi: 10.1186/1471-2202-12-7 (PMC3031267; doi:10.1186/1471-2202-12-7)

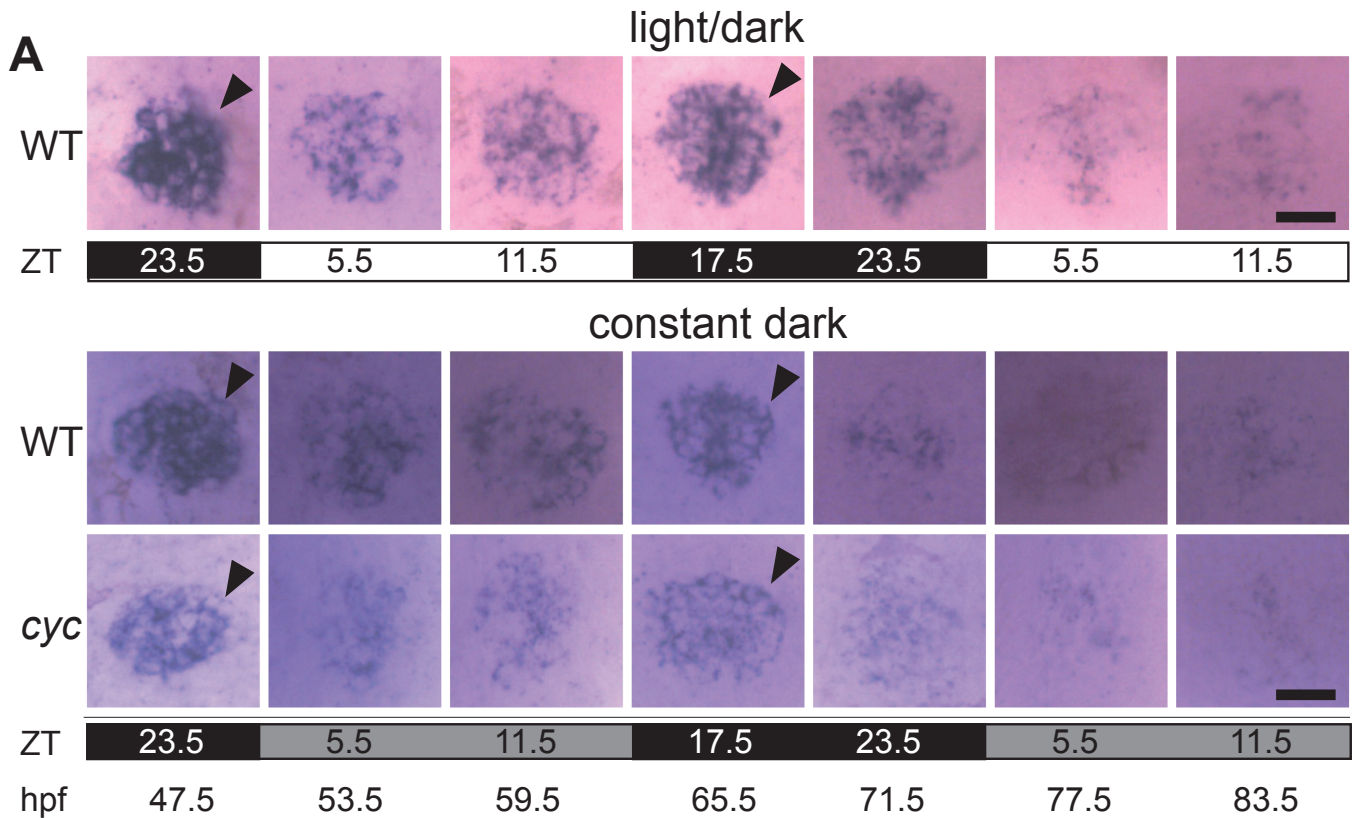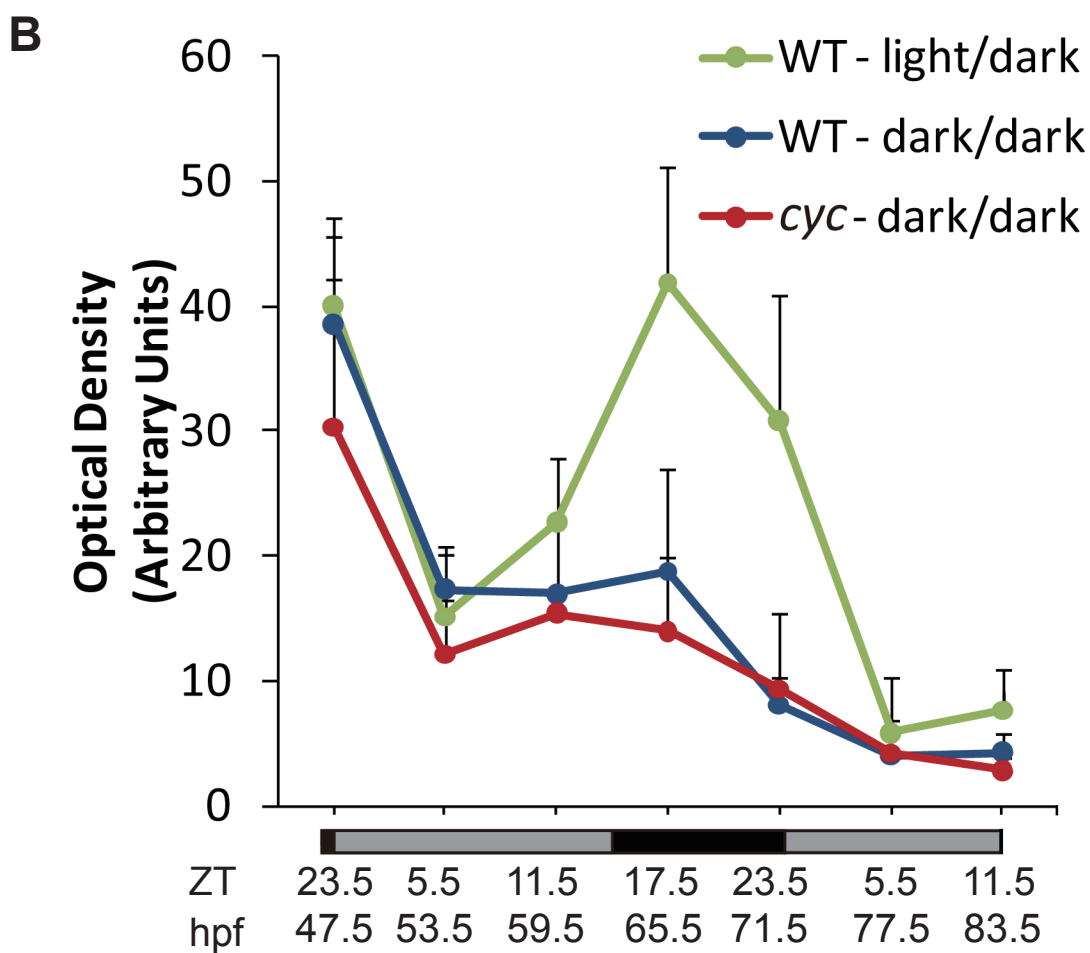

Supplement: Additional File 2 — Two days in a L/D cycle is not sufficient to initiate robust circadian cycling of aanat2 expression. Embryos were raised in a 14:10 h L/D cycle. At 47.5 hpf, ZT 23.5, a set of embryos was transferred to constant dark, constant temperature conditions. Embryos were fixed at the indicated stages and ZT and (A) processed for WISH for expression of aanat2 and (B) the WISH signal was quantified. Note the time of peak (closed arrowheads) expression is similar between the cyc embryos and their WT siblings. All images are dorsal views, anterior to the top. For the samples in a L/D cycle, position within the photoperiod is indicated by ZT and light conditions by the white (light period) and black (dark period) bars. For the constant dark samples, the original L/D cycle is indicated by the ZT and the black (original dark period) and dark grey (original light period) bars. Experiment was repeated two times with similar results, and representative images from one of the experiments are shown. Scale bars = 20 μm. [file 1471-2202-12-7-S2.PDF]
