# Supplementary material for: Circadian rhythms in the pineal organ persist in zebrafish larvae that lack ventral brain
Source: BMC Neurosci. 2011 Jan 13;12:7. doi: 10.1186/1471-2202-12-7 (PMC3031267; doi:10.1186/1471-2202-12-7)
Supplement: Additional File 3 — Comparison between larva processed for WISH with antisense and sense probes reveals low background staining. Embryos were raised in a 14:10 h L/D cycle and fixed and processed for WISH using aanat2 antisense or sense probe. Note that the sense probe produces no detectable signal, as it would recognize antisense mRNA, which should not be present. The brown regions are melanocytes in the skin, which have a natural pigment. All images are dorsal views, anterior to the top, with the pineal indicated (closed arrowheads). Position within the photoperiod is indicated by ZT and light conditions by the white (light period) and black (dark period) bars. Representative images are shown. Scale bar = 30 μm. [file 1471-2202-12-7-S3.PDF]

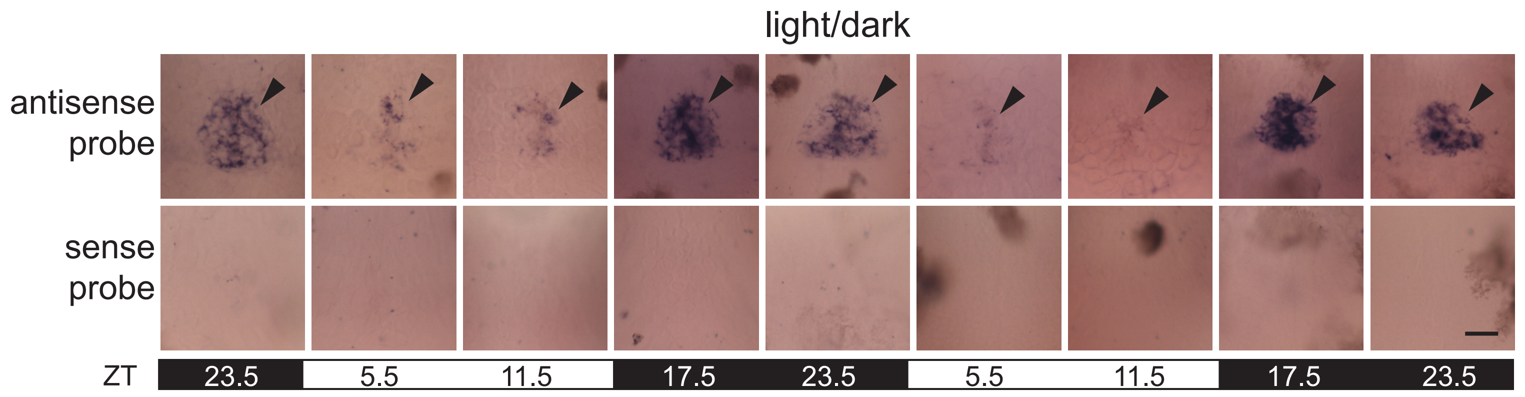
light/dark
antisense probe
sense probe
ZT
23.5
5.5
11.5
17.5
23.5
5.5
11.5
17.5
23.5
